# Urgent need for scaling up vaccine research on WHO priority fungal pathogens, authors’ reply

**DOI:** 10.1016/j.ebiom.2025.105621

**Published:** 2025-02-19

**Authors:** Mateusz Hasso-Agopsowicz, Angela Hwang, Maria-Graciela Hollm-Delgado, Isis Umbelino-Walker, Ruth A. Karron, Raman Rao, Kwaku Poku Asante, Meru Sheel, Erin Sparrow, Birgitte Giersing

**Affiliations:** a World Health Organization, Geneva, Switzerland; b Bridges to Development, Geneva, Switzerland; c Angela Hwang Consulting, Albany, CA, USA; d Department of International Health, Bloomberg School of Public Health, Johns Hopkins University, USA; e Hilleman Laboratories, Singapore; f Kintampo Health Research Centre, Ghana Health Service, Kintampo North Municipality, Ghana; g Sydney School of Public Health, Faculty of Medicine and Health, The University of Sydney, Australia

We appreciate the opportunity to respond to the letter “*Urgent Need for Scaling Up Vaccine Research on WHO Priority Fungal Pathogens*”. We acknowledge the critical importance of fungal pathogens, and the challenges associated with addressing them in global health. We also recognize the urgent need for increased basic research to understand the feasibility of development and use of vaccines against fungal pathogens.

It is important to understand, however, that the absence of fungal pathogens from our research priorities list is not because they have been overlooked, but rather due to the lack of vaccine candidates in clinical development. To focus on products with the greatest feasibility, our methodology limited the scope to pathogens with vaccine candidates in clinical development. Developing fungal vaccines faces significant challenges, including difficulty identifying fungal-specific vaccine targets and inducing protective immunity, particularly in immunocompromised individuals who are most at risk of severe fungal infections.^1^ At the time of our study, we found no fungal vaccines in clinical development, and to date, no fungal vaccine candidate has reached proof-of-concept stage. This remains a major scientific and clinical barrier that warrants further exploration.

Fungal vaccines are also challenging to prioritize due to the lack of data about the burden of fungal infections. Such data are crucial to enable evidence-based prioritization within the WHO’s vaccine research agenda. At the time our review was conducted, insufficient robust global data were available to estimate the burden of fungal infections.

In conducting our prioritization exercise, we applied a systematic, pathogen-agnostic approach based on current evidence and the values of regional stakeholders. The absence of fungal pathogens from the pathogen priority list for vaccines research and development reflects the current evidence. While we do not agree with the call to re-evaluate this priority list, we fully agree with the letter writers’ call for greater advocacy for and investment in addressing fungal pathogens. Such a call to action was issued by WHO in October 2022, with WHO's fungal priority pathogens list to guide research, development and public health action.^2^

Thank you for the opportunity to engage in this important discussion.

Sincerely, Mateusz Hasso-Agopsowicz

## Declaration of interests

Angela Hwang and Kwaku Poku Asante declare on their ICMJE forms consulting fees and support for attending meetings related to this research. The remaining authors declare no conflict of interest.
